# Antidepressants as emerging contaminants: Occurrence in wastewater treatment plants and surface waters in Hangzhou, China

**DOI:** 10.3389/fpubh.2022.963257

**Published:** 2022-08-11

**Authors:** Yuan Chen, Junlin Wang, Peiwei Xu, Jie Xiang, Dandan Xu, Ping Cheng, Xiaofeng Wang, Lizhi Wu, Nianhua Zhang, Zhijian Chen

**Affiliations:** Zhejiang Provincial Center for Disease Control and Prevention, Hangzhou, China

**Keywords:** antidepressants, occurrence, wastewater treatment plants, surface water, drinking water

## Abstract

**Aims:**

Antidepressants have aroused wide public concern due to their widespread presence in water and their harm to human health and environment. This study was designed to evaluate the contribution of wastewater treatment plants (WWTPs) to the presence of antidepressants in the surface water.

**Methods:**

Data was evaluated by analyzing water samples collected from the influent, effluent, upstream and downstream of the WWTPs on the rivers of interest in Hangzhou, Zhejiang Province, China. Besides, the study also assessed the impact of the release of antidepressants from WWTPs to the surface water on the drinking water. An automatic solid-phase extraction combined with ultra-high performance liquid chromatography-tandem triple quadrupole mass spectrometry (UPLC-MS/MS) was used to detect antidepressants.

**Results:**

The most abundant compound was venlafaxine, followed by citalopram, sertraline, and fluvoxamine with concentrations between 0.6 and 87 ng/L. Antidepressants showed maximum concentrations at the effluent outlets of the WWTPs, and greater concentrations were found downstream than upstream of the WWTPs in Qiantang River. The results of source water and finished water showed that the detection concentration was lower than the detection limit of the method.

**Conclusions:**

The less impact of the release of antidepressants from WWTPs to the surface water on the drinking water was identified. Nevertheless, these compounds were hardly removed by wastewater treatment processes. Thus, their risks deserve close attention.

## Background

Antidepressants are a group of drugs used to treat psychiatric disorders (1), which can be classified as tricyclic antidepressants (TCA), serotonin reuptake inhibitors (SSRIs), serotonin-norepinephrine reuptake inhibitors (SNRIs), and monoamine oxidase inhibitors (MAOIs) according to their mechanisms of action (2). The most frequently found psychiatric drugs were antidepressants such as fluoxetine, carbamazepine, citalopram, sertraline, and trazodone in concentrations of up to 2.0 ng/ L (3). In recent years, antidepressants have acquired much attention because of their occurrence in the environment water and aquatic organisms, as well as their potential harm to ecosystems and human wellbeing. Some research (4–7) suggests that the toxicological effects of antidepressants in different organisms, primarily fish, aquatic plants and mammals included changes in weight, pathological changes in brain, heart, and kidney, decrease in sperm dose (8). Antidepressants are introduced to the environment because of a variety of human activities. Much of the active ingredients in antidepressants which acted on humans are excreted to the environment, even some drugs are discarded without being used. (8) Most of them do not have 100% removal efficiency in WWTPs (9).

China has the largest population and the most pharmaceutical manufacturers in the world. The data from some major Chinese cities show that the total annual cost of antidepressants in 2014 was 2.679 billion RMB. The consumption sum of antidepressants was increased by years in 11 hospitals from Zhejiang during 2013–2017, and increased from 3,235,200 RMB in 2013 to 4,569,100 RMB in 2017; The top 3 drugs by consumption sum were fluoxetine,duloxetine,and venlafaxine; consumption sum of escitalopram accounted for a larger increase (proportion ration increased from 8th place in 2013 to first place in 2017). In China, antidepressants have been detected in Huangpu River, Dongting lakes, and Beiyun River (10, 11) with concentrations ranging from 3.2 to 22.9 ng/L. Occurrences of these antidepressants in the ambient river, water environment have been reported in the USA, France, Brazil, Canada, Australia, and the Czech Republic. Antidepressants were detected in rivers in concentrations ranging from 0.2 to 641 ng/L (12–16). As reported in the literature, antidepressant drugs have been found in several water bodies spanning different continents with a concentration ranging from Limit of Detections (LODs) to 326 ng/L in the influent and LODs to 374 ng/L in the effluent of 19 wastewater plants around metropolitan areas in Europe, Asia, America as well as Africa (17–20). Moreover, the concentrations of citalopram and fluoxetine detected in drinking water in the UK were ranging from 2.26 and 2.80 and 0.27 ng/L, respectively (21).

Zhejiang is one of the largest commercial and financial centers in China. Hangzhou, the capital of Zhejiang province, is a city with a population of more than 10 million. The dense population makes Hangzhou a large antidepressants consumption region in China as well as in the whole world. Qiantang River is the largest river in Zhejiang Province. It starts from Majinxi, the upstream of Qujiang River in the south. The river is 522.22 km long from its source, flowing through the southern part of Anhui province and Zhejiang Province, with a basin area of 55,058 square kilometers, and emptying into the East China Sea through Hangzhou Bay. Qiantang River system is a representative water body that has been impacted by urbanization in Hangzhou, Zhejiang province, which receives continuous effluents from industrial and/or sewage treatment plants. It has undergone serious deterioration in water quality in the recent years. Water and clean habitat are fundamental human needs. The Hangzhou section of Qiantang River is an important source of drinking water in Hangzhou. At the time of the study, the status of antidepressants in the water was unknown. In view of this, the aim of this study was to (i) examine the occurrence and distribution of antidepressants in the aquatic environment of Hangzhou and (ii) elucidate possible sources of those target antidepressants. The resulting data will be useful in enriching research on emerging pollutants in aquatic environments.

## Methods

### Study sites and sampling

**Figure 1:**
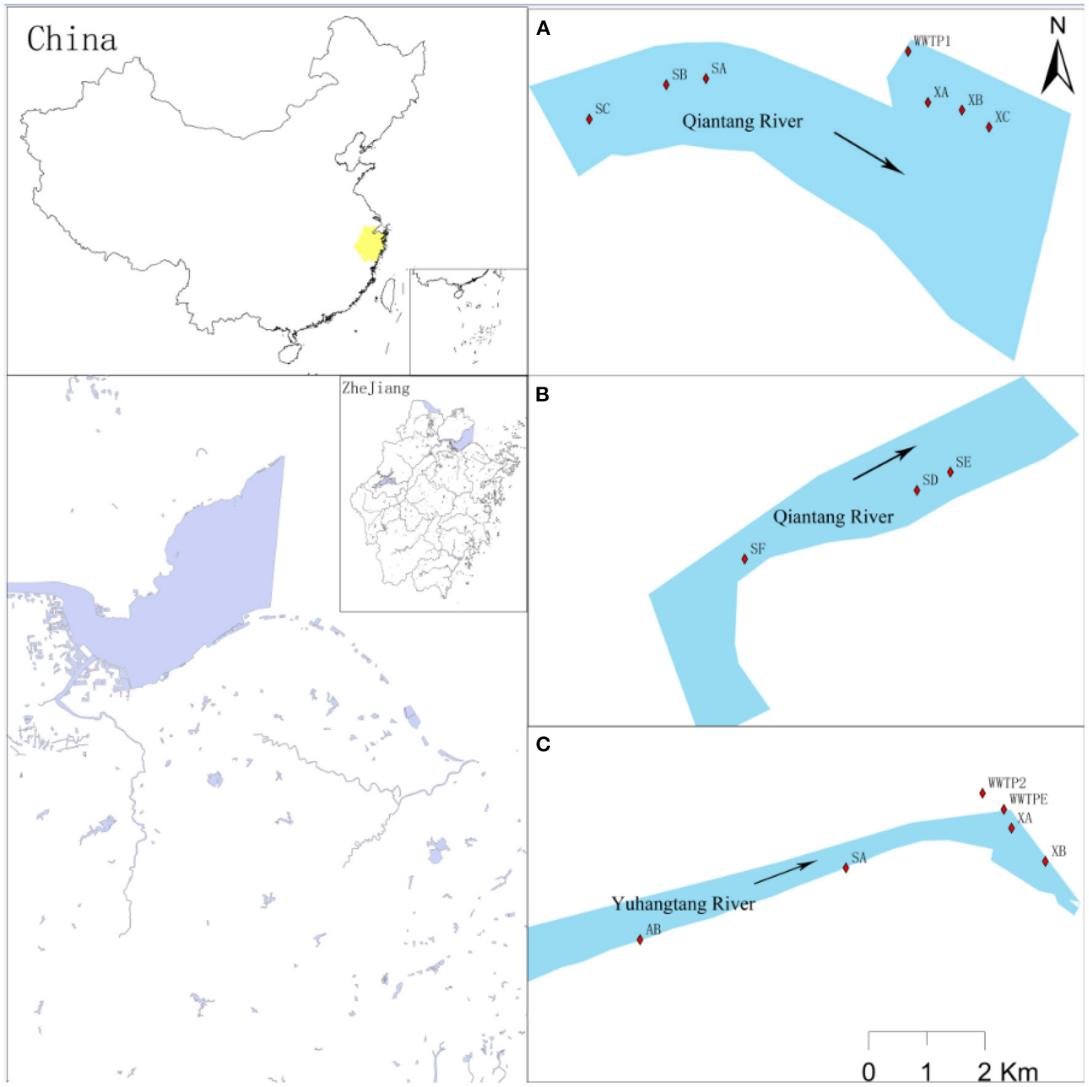
**(A)** Sampling locations in Qiantang River and WWTP 1. S(A,B,C) upstream of WWTP1, X(A,B,C) downstream of WWTP1. **(B)** Sampling locations in Qiantang River. S(D,E,F) locations of source water from the three DWTPs. **(C)** Sampling locations in Yuhangtang River and WWTP 2. S(A,B) upstream of WWTP2, X(A,B) downstream of WWTP2. WWTPE effluent of WWTP2

We collected one samples of sewage from the inlet and outlet of the two main WWTPs in Hangzhou. The sewage was collected by a 2.5 L deep water sewage collector. Sample volumes of 1 L were added into pre-cleaned glass bottles. Since Qiantang River is the main receiving water body for treated and untreated wastewater in Hangzhou *via* tributaries and the sewerage systems, antidepressant pollution in the Qiantang River could potentially be widespread, especially near the WWTPs. The six grabbed samples (XA, XB, XC, SA, SB, SC) were taken from the main stream of the Qiantang River in late November 2020 (sampling points are georeferenced in Figure 1A). River waters were collected 0.5-m deep near the margin of each river (~50 cm) using a mat high-density polyethylene bottle pre-washed with ultrapure water. Grab surface water samples were collected at a 0.5-m depth in Qiantang River, ~0.5, 1, 2 km upstream and downstream of the WWTP1 effluent position of the river. The three samples (SD, SE, SF) were source waters from the three Drinking Water Treatment Plants (DWTPs) located in the Qiantang River. These samples were taken on a boat at 1.0-m depth in the Qiantang River using an amber glass bottle about 20 km upstream of WWTP1 (sampling points are georeferenced in Figure 1B). Three samples of source water and three samples of finished water from three water plants on Qiantang River as the source water were collected and tested for eight kinds of antidepressants. Sample volumes of 1 L were added into pre-cleaned glass bottles. Before collection, the bottles were pre-rinsed with sample water again. All glassware used in this study was thoroughly washed with detergent at the laboratory.

The other five samples (YSA, YSB, YK, YXA, YXB) were collected at the beginning of November 2020 (sampling points are georeferenced in Figure 1C). These samples were taken at 0.5-m depth in Yuhangtang River and 1, 2 km upstream and downstream of the ChengXi wastewater treatment plant effluent, respectively. One liter of the sample water was collected in a pre-cleaned amber glass bottle. All these samples were stored in dark at 4°C for <24 h and were extracted as soon as possible in order to minimize the degradation. Once in the laboratory, all the samples analyzed in this study were adjusted to pH value 2.5 with hydrochloric acid and then filtered with GF/C (Whatman) glass fiber filter. During sample collection, a global positioning system (MG 758; Un Strong) was used to locate the sampling sites.

### Chemicals and reagents

Standards of eight antidepressants, paroxetine (Par), citalopram (Cit), and clomipramine (Clo) were purchased from Chiron (Norway). Amitriptyline (Ami), fluoxetine (Flu), venlafaxine (Ven), Sertraline (Ser) and trimipramine (Tri) were produced by the Shanghai Anpel Scientific Instrument Corporation. The physicochemical properties of the investigated compounds are shown in Table 1. The internal standards (IS) were obtained from Toronto Research Chemicals Dr. E. HPLC-grade methanol and acetonitrile were purchased from Merk (Darmstadt, Germany), and HPLC-grade formic acid was purchased from ACS Corporation (US). The ultra-pure water was produced by Milli-Qunit (Millipore, USA). Other chemicals and solvents were of analytical grade provided by Shanghai Anpel Scientific Instrument Corporation, Shanghai Lingfeng Reagent Corporation, Guoyao Corporation, and Guangdong Guanghua Reagent Corporation.

**Table 1:** List of the optimized MRM parameters and the selection of IS.

**Compounds**	**Retention**	**Precursor**	**Cone**	**Quantification**	**Collision**	**Identification**	**Collision**	**Selection**
	**time (min)**	**ion (*m/z*)**	**voltage (V)**	**trace**	**energy (eV)**	**trace**	**energy (eV)**	**of IS**
Amitriptyline	10.61	278	45	190.9	25	232.9	20	Amitriptyline-D3
Venlafaxine	7.88	278.2	20	120.8	35	260	10	Venlafaxine-D6
Duloxetine	10.38	298	30	153.8	5	183	20	Duloxetine-D7
Sertraline	11.16	305.9	35	158.7	30	274.7	10	Sertraline-D3
Fluoxetine	10.96	310.3	30	148.5	5	43.8	5	Sertraline-D3
Fluvoxamine	10.19	319.2	15	145	40	199.9	25	Sertraline-D3
Citalopram	9.21	325.2	10	233.9	25	262	20	Citalopram-D6
Paroxetine	9.88	329.9	10	150.9	25	192	35	Citalopram-D6
Amitriptyline-D3	10.59	281	45	191	25			
Venlafaxine-D6	7.85	284.1	20	121	35			
Duloxetine-D7	10.38	305.1	30	154	5			
Sertraline-D3	11.15	308.9	35	158.8	30			
Citalopram-D6	9.18	331	10	233.9	25			

#### Solid phase extraction procedure

The solid phase extraction (SPE) procedure referred to EPA 1694 extracted under acidic (pH 2.0 ± 0.5) conditions for determination of compounds upper than 200 kinds by auto-solid phase extraction. Samples were first filtered by GF/C glass filter, and then 125 mg EDTA-Na4 was dissolved to 250 ml filtered liquid and adjusted to pH 2.5. Next 10 ng IS was added. An Oasis HLB (500 mg, 6 ml) was used for concentration and purification. First, 6 ml methanol were used for activation SPE column and 6 ml ultra-pure water for equilibrium. Then, samples were loaded at 5 ml/min and washed using 5% methanol aqueous, and the SPE column was dried with nitrogen. Then, 10 ml methanol was used for elution, and the elution solvent was evaporated to 1.0 ml under a gentle nitrogen stream at 40°C and filtered using a glass filter to a 2 ml sample bottle for detection. The accuracy of the method was performed by sample spike at three concentration (10, 20, and 100 ng/L) in source water, the Methodological Validation data were listed in Table 2. Ultra-pure water with spike of eight antidepressants was detected for quality control. In addition, every kind of samples (source water, influent water, finished water) also detect with parallel samples and spike samples in every 10 samples.

**Table 2:** Regression equation, coefficient, limit of detect (LOD), limit of quantification (LOQ), recovery and relative standard deviation (RSD) of eight antidepressant drugs (*n* = 3).

**Compounds**	**Equation**	***r***	**LOD (ng/L)**	**LOQ (ng/L)**	**Spike at 10 ng/L**		**Spike at 20 ng/L**		**Spike at 100 ng/L**	
					**Recovery**	**RSD**	**Recovery**	**RSD**	**Recovery**	**RSD**
					**(%)**	**(%)**	**(%)**	**(%)**	**(%)**	**(%)**
Fluoxetine	*Y* = 1.11288*X* + 0.469408	0.99215	1.2	4	113.4	5.18	103.2	4.17	97.5	2.52
Paroxetine	*Y* = 0.113207*X* + 0.02586	0.99655	1.2	4	80.1	6.83	84.3	3.46	89.7	3.15
Citalopram	*Y* = 0.357815*X*-0.028035	0.99844	0.3	1	104.5	3.17	99.5	2.45	98.8	1.63
Sertraline	*Y* = 7.84844*X* + 0.362173	0.99882	1.2	4	95.1	3.09	97.8	2.88	101.1	1.27
Venlafaxine	*Y* = 0.495962*X* + 0.015519	0.99802	0.6	2	107.5	4.03	104.8	3.65	99.7	2.10
Amitriptyline	*Y* = 2.03739*X* + 0.051898	0.99898	1.2	4	94.1	3.12	96.7	2.16	99.4	1.08
Duloxetine	*Y* = 0.39801*X* + 0.091894	0.99723	3	10	104.3	3.89	102.5	3.22	98.6	2.02
Fluvoxamine	*Y* = 2.52171*X* + 0.26504	0.99668	1.2	4	86.8	5.62	89.3	4.66	92.1	3.17

#### Liquid chromatography–mass spectrometry

A 1-μl aliquot of each sample extract was separated using a Waters I-Class ultra-performance liquid chromatograph coupled to a Xevo TQ-S triple quadrupole mass spectrometer. A Waters UPLC Cortecs C18 reversed-phase column (150 mm × 3.0 mm, 1.6 μm) was used for the separation of compounds. The column was maintained at 30°C at a flow rate of 0.3 ml/min. Mobile phase A was 0.1% formic acid aqueous, and mobile phase B was 0.1% formic acid acetonitrile. The gradient (%B) is as follows: 0~0.5 min, 5%; 10 min, 35%; 16 min, 60%; 18 min, 75%; 19~20 min, 100%; and 20.1~23.5 min, 5%. To get the best detection signal for all basic analytes, the mass spectrometer was operated in electro-spray ionization (ESI) positive ion mode and multiple reaction monitoring (MRM) transition mode. Following the selection of the parent ions, daughter ions were obtained at a series of collision energies and selected according to the fragmentation that produced a useful abundance of fragment ions. The most abundant daughter ion was used for quantification and the second most abundant daughter ion for reliable identification. The following optimized parameters were used for the quantification of all compounds: drying gas temperature, 500°C; drying gas flow, 800 L/h; cone gas flow, 150 L/h; capillary voltage (+), 0.5 kV. The optimal LC-MS/MS parameters chosen for the identification and quantification of the eight antidepressants and five internal standards are listed in Table 1.

### Statistical analyses

The sampling distributions were labeled with ArcGIS10.2. Two parallel samples were taken from each sampling point, and the mean values were taken for analysis. Microsoft Excel 2013 was used to make tables and figures.

## Results and discussions

### Occurrence of antidepressants in influent and effluent of WWTPs

**Figure 2:**
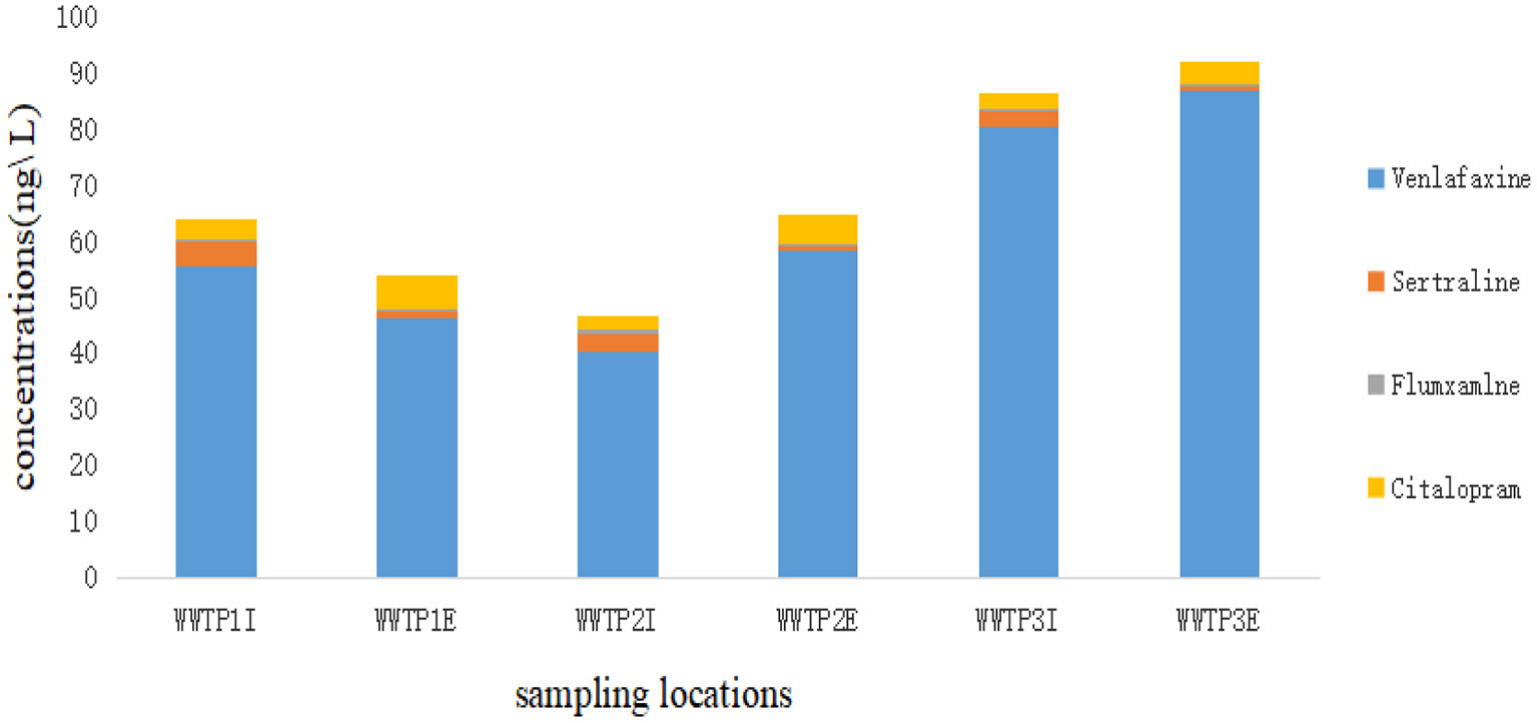
Concentration of antidepressant drugs in WWTPs.

Six sampling sites were selected covering the Qige and Chengxi WWTPs. These include the import and export sewage from the first and third phase of Qige and west of the city sewage treatment plants as shown in Figure 2. Four kinds of antidepressants were detected. Venlafaxine was detected in the highest concentrations (50.25 ± 1.05 ng/L) in all wastewater samples [median ± interquartile range (IQR)]. It was found in much higher concentrations than other antidepressants. Citalopram, sertraline, and fluvoxamine came next. The highest concentration of citalopram and sertraline was 5.75 and 4.25 ng/L, respectively, detected in the Import and export of WWTP1. SSRIs have been found in several water bodies spanning different continents,the average influent concentration of SSRIs in 11 WWTPs around metropolitan areas in the Pacific Coast and the Caribbean Lowlands was 600 ng/L and the average effluent concentration was 100 ng/L (8). In Canada, citalopram has been detected in five WWTPs, with concentrations in the influent and effluent ranging from 136 to 326 and 86 to 223 ng/L, respectively (14). Venlafaxine has been found in the United States at concentrations ranging from 210 to 220 ng/L in two WWTPs (22). In Beijing, China, venlafaxine was detected in three different WWTPs at concentrations of 31.8, 63.7, and 30.3 ng/L, respectively (23). The main types of antidepressants detected in this study were consistent with those reported in the literature above, and the concentrations were lower than those reported in other countries while consistent with domestic reports. The concentrations of antidepressants in the effluent were close to or even higher than those in the influent. The summary of concentrations of antidepressant drugs in the Import and export of WWTPs was listed in Table 4, the results in this study were consistent with those reported domestic and international, demonstrating that these compounds were hardly removed by wastewater treatment processes? Thus, their risks deserve close attention.

At present, there is little research on antidepressants in WWTPs in China. Only one report cited above was found. This study expanded data on antidepressant levels in WWTPs.

### Occurrence of antidepressants in Qiantang River and Yuhangtang River

**Figure 3:**
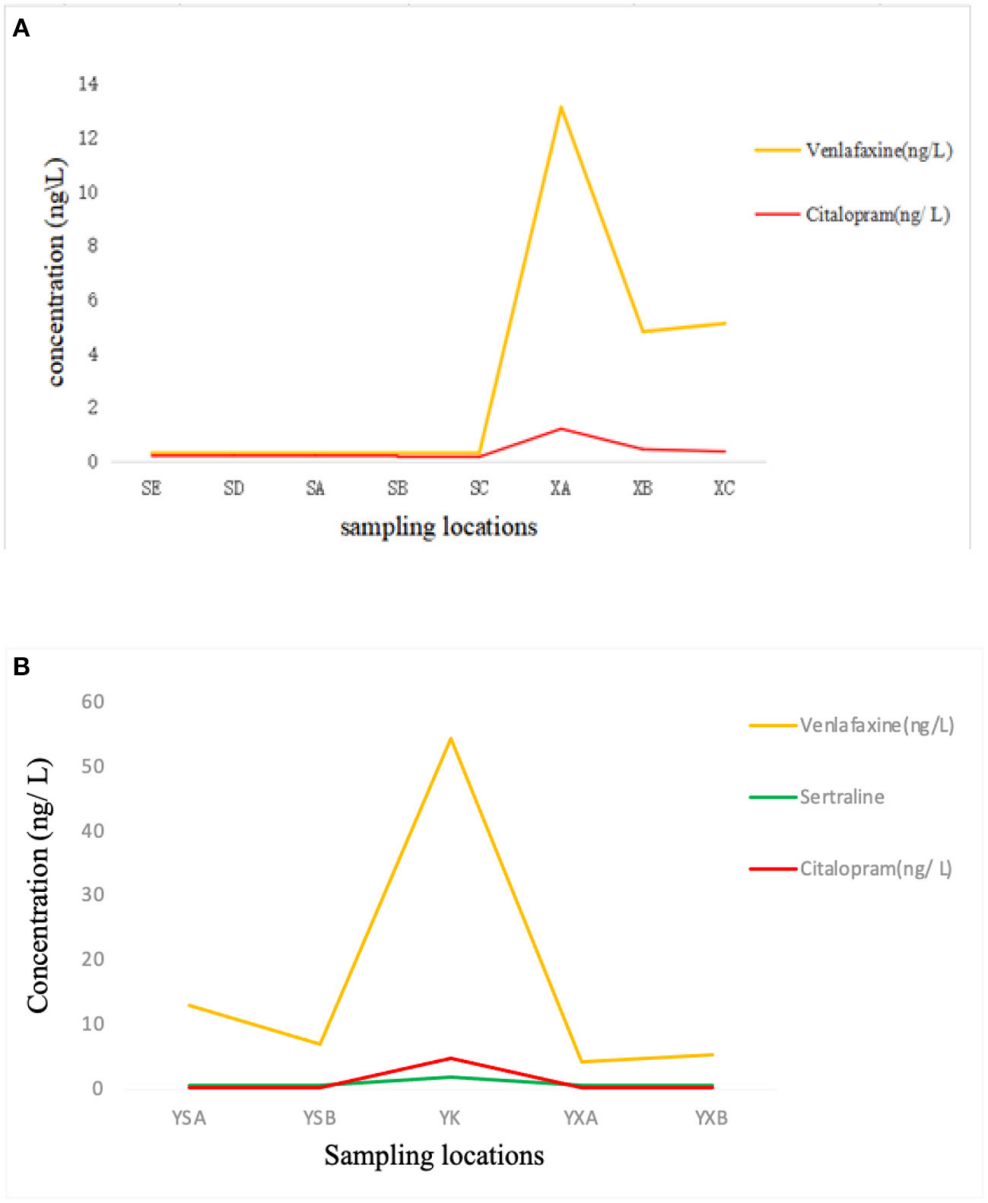
**(A)** Concentration of antidepressant drugs in Qiantang River. **(B)** Concentration of antidepressant drugs in Yuhangtang River.

Surface water was collected ~1.0, 1. 5, 2.0 km upstream and downstream of the two WWTPs' sewage draining exits. The sample point locations are shown in Figure 1. The results are shown in Figure 3. Three kinds of antidepressants were detected, and the levels were generally in the range of a few tenths to tens of ng/L. Venlafaxine was detected in all samples with the highest levels in the sewage draining exit of WWTP2, where the concentration was 54.2 ng/L. The venlafaxine level is higher than Huangpu River, Dongting River, and Beiyun River in China, which range from 1 to 22.9 ng/L (10, 11, 30), but lower then Leca River in Portugal (3) and Guayllabamba River in Ecuador, where venlafaxine was reported in concentrations of up to 55,000 ng /L (31). The summary of concentrations of antidepressant drugs in WWTPs in other countries was listed in Table 3. Citalopram was also detected in the two rivers, with concentrations ranging from <LODs to 4.8 ng/L, with the highest levels in the sewage draining exit of WWTP2. Sertraline was detected only in Yuhangtang River, with concentrations ranging from <LODs to 1.9 ng/L. The trend of antidepressants concentration at different sampling points in the same river is shown in Figure 3. The concentration of antidepressants was maximum at the sewage disposal outlet of the WWTPs and then decreased progressively along the upper and lower reaches of the river. To better understand the effect of WWTPs on the environment, we compared the concentrations of antidepressants upstream and downstream of rivers. The concentrations of citalopram and venlafaxine were higher downstream of Qiantang River than upstream, the sampling locations in the abscissa follow the river flow from upstream to downstream of Qiantang River was shown in Figure 3A. Significantly, an increase tread was observed in the level of antidepressants from upstream to downstream of the WWTPs along the river of SE-SD-SC-SB-SA-XA-XB-XC, indicating WWTPs are sources of antidepressants into the environment. The WWTPs might be source of river basin pollution. While the concentration of venlafaxine was higher upstream of Yuhangtang River than downstream. The other two antidepressants concentrations were similar upstream and downstream in Yuhangtang River which was shown in Figure 3B. There are two sewage outlets in Yuhangtang River, one of which has been identified by us, and the other is upstream of the sewage outlet, but the location is not clear. This may be the reason for higher concentrations of venlafaxine upstream of Yuhangtang River than downstream. The other reason could be the mixing effect of passing freight vessels on Yuhangtang River. The summary of concentrations of antidepressant drugs in surface waters in other countries was listed in Table 4. Antidepressants discharged from WWTPs into the surface water, then through the mixed dilution of waterbody and human activities. The decay process of the target substance in river is complicated.

**Table 3:** Summary of concentration of antidepressant drugs in WWTPs.

**Anti**	**Location**	**Source**	**Concentration**		**Reference**
**depressants**			**(ng/L)**	
			**Influent**	**Effluent**	
Fluoxetine	Costa Rica	WWTP	~60	~100	(24)
	Canada	WWTP(5)	9~26	7.6~20	(14)
	China	WWTP(3)	0.6~4.25	0.3~1.05	This study
Sertraline	USA	WWTP		3	(25)
	Canada	WWTP(3)	12~26	8.1~16	(14)
	China	WWTP(3)	0.6~1.5		This study
Citalopram	Canada	WWTP(5)	136~326	131~223	(14)
	China	WWTP(3)	0.6~3.2	0.3~7.6	This study
Imipramine	Spain	WWTP		3	(26)
	China	WWTP		10.9	(23)
Venlafaxine	Portugal	WWTP	39.4~66.7	327~374	(27)
	USA	WWTP		210~220	(22)
	China	WWTP(3)	40.25~80.4	21~87	This study

**Table 4:** Summary of concentration of antidepressant drugs in surface waters.

**Anti**	**Location**	**Source**	**Concentra-**	**Reference**
**depressants**			**tion (ng/L)**	
Fluoxetine	Brazil	Santos Bay	0.58	(27)
Citalopram	Czech Republic	Blanice River	24	(16)
	China	Qiantang River	~4.8	This study
Venlafaxine	China	Beiyun River	22.9	(28)
	Portugal	Leça River	641	(3)
	South Africa	Jukskei River	0.2~4.0	(29)
	Portugal	Lis River	159	(27)
	China	Qiantang River	~54.2	This study
Sertraline	China	Huangpu River	3.2	(30)
	China	Yuhangtang River	~1.9	This study

### Occurrence of antidepressants in source water and finished water

We collected three samples of source water and three samples of finished water from three water plants on Qiantang River as the source water and tested them for eight kinds of antidepressants. The results showed that the detection concentration was all lower than the detection limit of the method. The levels were significantly lower than those reported in drinking waters in other countries. In the UK, citalopram and fluoxetine have been detected in drinking water at concentrations of 2.26–2.80 and 0.27 ng/L, respectively (21). The maximum concentration of citalopram in Danube-derived tap water from the Budapest metropolitan region (Hungary) was 0.590 ng/L (1). Only trace amounts of antidepressants including citalopram (up to 1.5 ng/L), sertraline (up to 3.1 ng/L), and venlafaxine (up to 1.9 ng/L) were detected in tap water in Warsaw (Poland) (32). Antidepressants discharged from WWTPs into the river, through the mixed dilution of waterbody, the concentration in the downstream gradually decreased. The concentration of antidepressants in the upper reaches of Qiantang River which as the source of drinking water was low, thus indicating that the health risk of these substances in Qiantang River as a source of drinking water might be low.

### Strengths and limitations

At present, there is only one report on antidepressants in WWTPs in China. Expanded data on antidepressant levels in WWTPs is needed. The resulting data will be useful in enriching research on emerging pollutants in WWTPs and aquatic environments. However, our study also has some minor shortcomings. For example, the number of samples collected from WWTPs did not consider sampling at different time intervals, resulting in a small number of overall samples. However, this study has a certain indicative value as a suggestive study. Further consideration will be given to improving the design in subsequent studies.

## Conclusions

In the present study, we investigated the occurrence of eight antidepressants in the inlet and outlet of two WWTPs and the upstream and downstream of their sewage river. Three samples of source water and finished water were collected from three water plants on Qiantang River as the source water and tested for eight kinds of antidepressants. It is worth mentioning the less impact of the release of antidepressants from WWTPs to the surface water on the drinking water. Nevertheless, the concentrations of antidepressants in the effluent were even higher than those in the influent, demonstrating that these compounds were hardly removed by wastewater treatment processes. Thus, their risks deserve close attention.

## Data availability statement

The original contributions presented in the study are included in the article/supplementary material, further inquiries can be directed to the corresponding author.

## Author contributions

YC, PX, ZC, and XW made contributions to the conception and design of the study. YC, JX, DX, and PC collected the samples and cleaned the data. JW, LW, and NZ tested the samples. YC did the statistical analysis and drafted the article. All authors contributed interpreting the results and revised the draft critically.

## Funding

This study was supported by the Foundation of the Medical Scientific Research of Zhejiang Province (2020KY514, 2021KY621, 2022RC121, and 2022RC122).

## Conflict of interest

The authors declare that the research was conducted in the absence of any commercial or financial relationships that could be construed as a potential conflict of interest.

## Publisher's note

All claims expressed in this article are solely those of the authors and do not necessarily represent those of their affiliated organizations, or those of the publisher, the editors and the reviewers. Any product that may be evaluated in this article, or claim that may be made by its manufacturer, is not guaranteed or endorsed by the publisher.
